# Overexpression of miR-484 and miR-744 in Vero cells alters Dengue virus replication

**DOI:** 10.1590/0074-02760160404

**Published:** 2017-03-02

**Authors:** Juan Camilo Castrillón-Betancur, Silvio Urcuqui-Inchima

**Affiliations:** Universidad de Antioquia, Facultad de Medicina, Grupo Inmunovirología, Medellín, Colombia

**Keywords:** Dengue virus, microRNA, 3’ untranslated region, virus replication

## Abstract

**BACKGROUND:**

Dengue is considered one of the world’s most important mosquito-borne diseases. MicroRNAs (miRNAs) are small non-coding single-stranded RNAs that play an important role in the regulation of gene expression in eukaryotes. Although miRNAs possess antiviral activity against many mammalian-infecting viruses, their involvement in Dengue virus (DENV) replication remains poorly understood.

**OBJECTIVE:**

To determine the role of miR-484 and miR-744 in DENV infection and to examine whether DENV infection alters the expression of both miRNAs.

**METHODS:**

We used bioinformatics tools to explore the relationship between DENV and cellular miRNAs. We then overexpressed miR-484 or miR-744 in Vero cells to examine their role in DENV replication using flow cytometry, reverse transcriptase quantitative polymerase chain reaction (RT-qPCR), and western blotting.

**FINDINGS:**

We found several cellular miRNAs that target a conserved region within the 3′ untranslated region (3′ UTR) of the genome of the four DENV serotypes and found that overexpression of miR-484 or miR-744 inhibits infection by DENV-1 to DENV-4. Furthermore, we observed that DENV RNA might be involved in the downregulation of endogenous miR-484 and miR-744.

**CONCLUSION:**

Our study identifies miR-484 and miR-744 as two possible restriction host factors against DENV infection. However, further studies are needed to directly verify whether miR-484 and miR-744 both have an anti-DENV effect in vivo.

Dengue is considered one of the most important diseases transmitted by mosquitos, its incidence has increased at an alarming rate and it has become a public health problem over the last fifty years (Bhatt et al. 2013). Among the causes of this increase are social and demographic changes such as population growth and urbanisation together with the lack of programs for surveillance, prevention, and vector control. Dengue is transmitted by the bite of a mosquito (*Aedes aegypti* or *Aedes albopictus*) infected with one of the four dengue virus (DENV) serotypes, which are genetically and antigenically related viruses. DENV is a member of the family *Flaviviridae*, genus *Flavivirus*. The genome consists of a positive single-stranded RNA (ssRNA) of about 11 kb with a single open reading frame encoding one polyprotein that is processed by cellular and viral proteinases to form three structural and seven non-structural (NS) proteins (Chambers et al. 1990). DENV RNA is flanked by 5′ and 3′ untranslated regions (UTRs) that present high degrees of consensus sequences among the four serotypes. The 3′ UTR contains two secondary hairpin structures known as 3′-stem loops (3′-SLs) and a small hairpin that are important elements for viral replication/translation, and are the most conserved structures in *Flavivirus* RNAs. Cellular proteins such as the translation elongation factor-1 alpha (EF-1α) and the polypyrimidine tract-binding (PTB) protein are reported to interact with the 3′-SL, suggesting that both proteins may function as chaperones to maintain the RNA structure in a conformation that favours DENV replication (de Nova-Ocampo et al. 2002). Furthermore, Alvarez et al. (2008) described a sequence located upstream of the translation initiation codon in the 5′ UTR, complementary to a region present in the 3′-SL, designated as the 5′ and 3′ upstream AUG region (5’-3’UAR), which is also essential for RNA circularisation and thus for viral RNA synthesis by the viral RNA-dependent RNA polymerase (RdRp).

Since the discovery of miRNAs, a class of endogenous non-coding cellular RNAs of 18-25 nucleotides that binds to a complementary sequence in the 3′ UTR of target mRNAs to regulate target gene expression mostly at the post-transcriptional level, their potential application for the treatment of infectious diseases is being investigated (Ghosh et al. 2009). In this context, some miRNAs are involved in the host antiviral response, acting as factors with antiviral activity. For example, the miRNA let-7b inhibits Hepatitis C Virus (HCV) RNA expression and replication by targeting the host factor insulin-like growth factor 2 mRNA-binding protein 1 (IGF2BP1) (Cheng et al. 2013). The 3′ end of the Human immunodeficiency virus type 1 (HIV-1) mRNA is also reported to be a target for a group of miRNAs expressed in resting CD4+ T cells (miR-28, -125b, -150, -223, and -382) that may inhibit translation and cause a latent infection in these cells (Huang et al. 2007). Recently, six single miRNAs targeting the highly conserved regions of the DENV-2 genome were shown to efficiently inhibit virus replication (Xie et al. 2013). However, DENV infection significantly induced the expression of miR-146a, thereby facilitating viral replication by targeting tumour necrosis factor receptor-associated factor 6 (TRAF6) and diminishing interferon-β (IFN-β) production (Wu et al. 2013). Additionally, incorporation of miR-122-MRE (miRNA recognition element) confers an inhibitory susceptibility to miR-122 targeting the DENV replicon, suggesting that DENV can be engineered to exert the desired replication restriction effect for avoiding infection of vital tissues/organs (Lee et al. 2010). Recently it was reported that DENV infection up-regulates the expression of miR-30e* but overexpression of this miRNA simultaneously suppresses DENV replication by promoting IFN-β production (Zhu et al. 2014). Overexpression of miR-548g-3p suppresses the replication of all four DENV serotypes (Wen et al. 2015) and we have demonstrated that overexpression of synthetic miR-133a suppresses DENV-2 replication, possibly through regulating the expression of PTB, a miRNA-133a target, since its protein was found to be upregulated during DENV infection (Castillo et al. 2016). Furthermore, we found that the 3′ UTR of the RNA of all four DENV serotypes is targeted by miR-133a. However, members of the *Flavivirus* genus were recently reported to produce a subgenomic flavivirus RNA (sfRNA), a positive-sense non-coding RNA that accumulates to high levels in infected mammalian and insect cells (Lin et al. 2004). Interestingly, this sfRNA acts as an RNA interference (RNAi) suppressor in both insect and mammalian cells, inhibiting the RNase Dicer (Schnettler et al. 2012). This is why the discovery of cellular miRNAs recognising target sequences in the DENV genome would be vital, possibly leading to a treatment for dengue, for which there is no vaccine or therapeutic drug yet.

Although the biological functions of miR-744 and miR-484 are poorly understood, they appear to be highly conserved in vertebrates. The gene encoding miR-744 locates at chromosome 17p12 and is implicated in the cellular process leading to human disease development. The function of miR-744 depends on the cell type and it acts as either a tumour suppressor or a tumour promoter. The gene encoding miR-484 locates at chromosome 16p13 and is essential for cerebral cortex development. Both, miR-744 and miR-484 are mainly expressed in the brain, but they can also be found in the liver and blood. miR-484 was significantly differentially expressed in the serum of patients with early breast cancer versus healthy controls; however, no correlation could be established between miR-484 levels and the histopathological parameters of breast cancer (Zearo et al. 2014). miR-744 is suggested to play an inhibitory role in many cancers, including colon, breast, and gastric cancer. It was recently reported that miR-744 inhibits the growth, migration, invasion, proliferation, and metastasis of gastric cancer by targeting Bcl-2 protein expression (Chen & Liu 2016). Furthermore, a lower miR-744 expression level was reported in patients with hepatocellular carcinoma (HCC) and this was associated with HCC recurrence and prognosis (Tan et al. 2015). However, studies regarding the role, effect, and significance of miR-484 and miR-744 on viral infections are still insufficient. Therefore, the main objective of this study was to determine the role of miR-484 and miR-744 in DENV infection and to examine whether DENV infection alters the expression of both these miRNAs. While our results were obtained using Vero, a cell line highly susceptible to DENV infection, we have also observed that macrophages and dendritic cells, which are both targets of DENV infection, also express miR-484 and miR-744 (unpublished observations). Therefore, we explored the relationship between DENV and cellular miRNAs using bioinformatics tools. We then overexpressed miR-484 or miR-744 in Vero cells to test their role in DENV replication, and finally determined the effect of DENV infection and the effect of the 3′ UTR of DENV RNA on miR-484 and miR-744 expression in Vero cells. We observed that overexpression of either miR-484 or miR-744 diminishes the replication of all four DENV serotypes. Finally, infection and transfection of Vero cells with a plasmid construct encoding the 3′ UTR of DENV RNA resulted in downregulation of endogenous miR-484 and miR-744. This is one of the first studies to demonstrate the effect of two cellular miRNAs on regulating the replication of the 4 DENV serotypes. Our study thus helps us to better understand the relationship between host cells and DENV infection. Although additional studies are required to unambiguously confirm the function of miR-484 and miR-744, our preliminary data suggest that these miRNAs might play a role in DENV replication.

## MATERIALS AND METHODS

*Bioinformatics predictions* - For the computational analysis, we followed a strategy established in our laboratory (Castillo et al. 2016). Briefly, the sequences of DENV-1 to -4 were downloaded from GenBank, and the free algorithm MicroInspector (www.ncbi.nlm.nih.gov/pmc/articles/PMC1160125/) was used to scan for possible targets of human miRNAs collected in miRBase. Only those target sites common to the four reference sequences were selected. The RNAhybrid program (http://bibiserv.techfak.uni-bielefeld.de/rnahybrid/) was used to verify the findings of MicroInspector.

*Cell lines* - Mosquito C6/36 HT cells obtained from the ATCC were cultured as previously described (Castillo et al. 2016). Vero cells (CCL-81) were grown in Dulbecco’s modified Eagle medium (DMEM) supplemented with 10% heat-inactivated foetal bovine serum (FBS), 1% L-glutamine, 1% vitamins, 1% non-essential amino acids, and 1% Penicillin/Streptomycin (Sigma-Aldrich Chemical Co, St. Louis, MO, USA), at 37ºC with 5% CO_2_.

*Viral stocks and titration* - The reference strains of DENV-1, DENV-2 New Guinea C (NGC), DENV-3, and DENV-4 were provided by the Centers for Disease Control (CDC, CO, USA). Viral stocks were obtained by inoculating a monolayer of C6/36 HT cells in a 75-cm^2^ tissue culture flask with the virus at a multiplicity of infection (MOI) of 0.05 diluted in 1 mL of L-15 medium supplemented with 2% FBS. After 3 h of adsorption, 10 mL of L15 medium supplemented with 2% FBS was added and the cells were cultured for five days at 34ºC without CO_2_. The supernatant was then removed from the cells and centrifuged for 5 min at 400 *x g* to pellet the cell debris. The supernatant was aliquoted and stored at -70ºC for future use. Clinical isolates of DENV-1 (strain Bga-07), DENV-2 (strain 109-05), and DENV-4 (strain Bga-06) were obtained from patients with dengue haemorrhagic fever in Antioquia, Colombia (kindly provided by Dr FJ Díaz, Grupo Inmunovirología, Facultad de Medicina, Universidad de Antioquia) and used to clone the DENV 3′ UTR (Castillo et al. 2016). Virus titration was performed by flow cytometry, as previously described (Castillo et al. 2016). Briefly, C6/36 HT cells were seeded in 12-well plates and cultured overnight at 34ºC without CO_2_. They were then infected with 10-fold serial dilutions of the virus, and at 24 h post-infection (hpi) they were harvested and resuspended in PBS. For flow cytometry analyses, the cells were fixed using a Fixation/Permeabilisation buffer (eBioscience, San Diego, CA, USA), centrifuged and washed twice with PBS, and stained with the monoclonal antibody 4G2 (kindly provided by Dr P Desprès, Institut Pasteur, Paris) in a final volume of 100 mL. As the secondary antibody, fluorescein isothiocyanate (FITC)-labelled goat anti-mouse IgG antibody (Invitrogen, Life Technologies, CA, USA) was used. The cells were analysed with a FACScan flow cytometer using the FACSdiva software. The percentage of infected cells in each sample and the total number of cells seeded per well were used to calculate the final virus titre.

*Generation of pGUD plasmid constructs* - pGUD plasmids containing the 3′ UTRs of DENV-1, -2, or -4 downstream of green fluorescent protein (GFP) in the pEGFP-C1 (Clontech, CA, USA) construct were previously described (Castillo et al. 2016). Briefly, the 3′ UTRs of the three DENV serotypes were amplified by polymerase chain reaction (PCR) from viral RNA obtained from infected cell culture supernatants using specific primers (forward: 5′ GAATTCG**TAG**GTGCGGCTCATTGATTGGGCTAAC 3′ and reverse: 5′ GTCGACGAACCTGTTGATTCAACAGCACC 3′). A stop codon (indicated in bold in the 5′ end of the forward primer), and a restriction site for EcoRI and SalI (underlined) at the 5′ end of the forward and reverse primers respectively, were incorporated during amplification. The PCR products were purified and cloned into the pEGFP-C1 construct, using the EcoRI and SalI enzymes (Thermo Scientific, NH, USA). The constructs generated were designated pGUD1, pGUD2, and pGUD4 for the DENV-1, DENV-2, and DENV-4 3′ UTR respectively. We were unable to amplify the 3′ UTR of DENV-3 RNA from the available DENV-3 isolates.

*Expression of miR-484 and miR-744* - Vero cells were seeded at a final concentration of 1.5 × 10^5^ cells/well in 24-well plates and transiently transfected with pGUD1, pGUD2, or pGUD4, at a final concentration of 0.5 µg/well, using Lipofectamine 2000 (Invitrogen) according to the manufacturer’s instructions. Alternatively, Vero cells were infected with each DENV serotype at an MOI of 3. In both cases, the cells were harvested at 8, 16, 24, 32, 48, and 72 hpi and total RNA extraction was performed using the Trizol reagent (Invitrogen) following the manufacturer’s instructions. The RNA concentration was measured using a NanoDrop spectrophotometer (Nano Drop Technologies, CA, USA). Reverse transcription of endogenous miR-484 and miR-744 in transfected and DENV-infected Vero cells was carried out with 10 ng RNA to produce cDNA using the TaqMan® MicroRNA Reverse Transcription Kit (Applied Biosystems, CA, USA). For miRNA reverse transcriptase quantitative-PCR (RT-qPCR), experiments were carried out using the Taqman microRNA Assay (Applied Biosystems) and TaqMan® Universal Master Mix II (Applied Biosystems), according to manufacturer’s instructions, and performed in triplicate on a Bio-Rad CFX96 real-time Detection System (Bio-Rad, CA, USA). The expression of both miRNAs was normalised to 18S rRNA, and the expression levels were determined via the comparative threshold cycle (Ct) method using 2^-ΔΔCt^. Three independent replicates were performed for each experiment.

*Analysis of the effect of miR-484 and miR-744 expression on DENV replication* - To evaluate the effect of miR-484 and miR-744 on DENV replication, Vero cells were seeded at a density of 2 × 10^5^ cells/well in 12-well plates. On the following day, the cells were transiently transfected with pEZX-MR03-miR-484 (*Homo sapiens* miR-484 stem-loop expression clone, cat. HmiR0264-MR03) or pEZX-MR03-miR-744 (*H. sapiens* miR-744 stem-loop expression clone, cat. HmiR0509-MR03) both obtained from GeneCopoeia, Inc. (Rockville, Maryland, USA), at a final concentration of 0.5 µg/mL using Lipofectamine 2000 according to the manufacturer’s instructions. A scrambled miRNA or empty vector (Cat. CmiR0001-MR03, Rockville, Maryland, USA) was used as the negative control. These constructs contain the GFP reporter gene facilitating the determination of transfection efficiency and the miRNA overexpression level by fluorescence microscopy. At 24 hpt, the cells were challenged with each DENV serotype separately at an MOI of 3. Alternatively, the cells were first challenged with each DENV serotype separately and after 24 h, they were transfected with pEZX-MR03-miR-484 or pEZX-MR03-miR-744 as described above. After 3 h of virus adsorption and shaking every 30 min, the medium was removed, the cells were washed twice with PBS, and the medium was replaced with DMEM supplemented with 2% FBS. The effect of either miRNA on DENV replication was assessed at 72 hpi by flow cytometry and RT-qPCR, and by western blot in the case of DENV-2. All experiments were performed in triplicate.

*Quantification of DENV infection by flow cytometry* - At the indicated time points post-infection, Vero cells were harvested and analysed by flow cytometry as described above. The infected cells were expressed as the percentage of infected cells over the total number of cells analysed. All the experiments were performed in triplicate.

*Quantification of DENV RNA copy number by RT-qPCR* - Viral RNA was extracted from culture supernatants using the QIAamp Viral RNA Mini Kit (Qiagen, Hilden, Germany), according to the manufacturer’s instructions. The viral copy number was determined by RT-qPCR using the following DENV-specific primers against the conserved sequences in the core gene of DENV RNA: forward: 5′ CAA TAT GCT GAA ACG CGA GAG AAA 3′, and reverse: 5′ CCC CAT CTA TTC AGA ATC CCT GCT 3′ (Castillo et al. 2016). Calculation of the genomic RNA copy number was performed based on a standard curve, as previously described (Sachs et al. 2011). All experiments were performed in triplicate.

*Viral protein detection by western blotting* - miRNA-transfected and DENV-2-infected cells were detached with trypsin and lysed using Lysis solution (Applied Biosystems). Total proteins were quantified using the BCA Protein Assay (Pierce, Thermo Scientific, NH, USA). Total protein (50 µg) was loaded onto an SDS-PAGE gel and then transferred to a nitrocellulose membrane after electrophoresis. A primary mouse monoclonal antibody against the DENV NS1 protein (Thermo Scientific) and a secondary anti-mouse IgG antibody conjugated with horseradish peroxidase (HRP; Santa Cruz Biotechnology, USA) were used for detection. Finally, the signals were detected using the chemiluminescence ECL^TM^ detection system (Pierce). Band intensities were quantified by densitometry using the image processing software ImageJ 1.49, freely available online.

*Statistical analysis* - The results are expressed as median with range. Statistical analyses were performed for triplicate experiments using the two-way ANOVA test. A p value less than 0.05 was considered significant.

## RESULTS

**Fig. 1:**
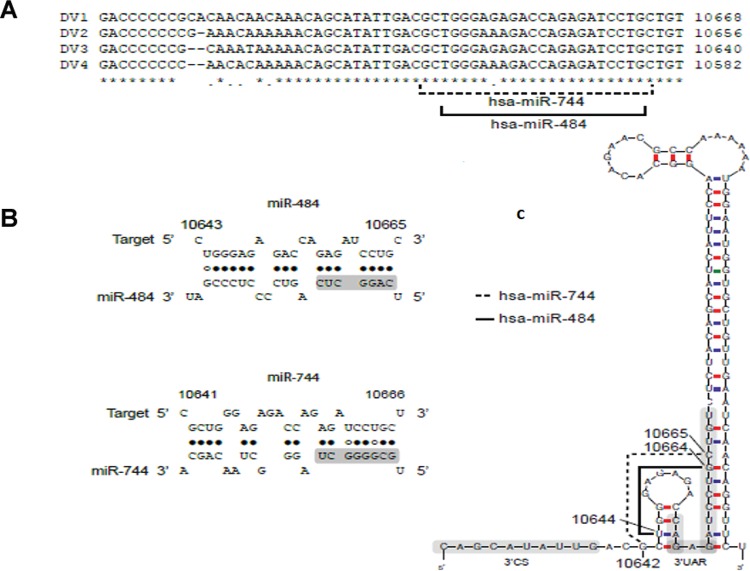
: the 3′ UTR of Dengue virus (DENV) RNA contains target sequences for cellular miR-484 and miR-744. (A) Alignment of a fragment of the 3′ UTR of the four DENV serotypes. The miR-484 and miR-744 target sites common to the four serotypes are indicated; (B) the target sites for miR-484 and miR-744 fulfil the seed sequence (shaded nucleotides, first nucleotides from the 5′ end of the miRNA). Structures predicted using RNAhybrid; (C) location of the miR-484 and miR-744 target sequences in the 3′-SL, CS, and 3′UAR regions of the 3′ UTR. The secondary structure of DENV RNA was predicted using the Mfold program (Zuker 2003). The sequences and position numbers in (B) and (C) correspond to DENV-1 RNA.

*DENV 3′ UTR contains potential miR-484 and miR-744 binding sites* - We were interested in determining whether any interaction occurs between DENV RNA and human host miRNAs, since it was previously reported that viral genomes can be targeted by human miRNAs. We therefore investigated whether the DENV 3′ UTR contains potential miRNA binding sites. The sequences of the 3′ UTRs plus 374 nucleotides of the coding region of NS5 of all four DENV serotypes were aligned. In total, 108 miRNAs for DENV-1, 80 for DENV-2, 94 for DENV-3, and 89 for DENV-4 were predicted to target the 3′ UTR using the MicroInspector software (data not shown). Since it is reported that the 3′ UTR sequence across all DENV serotypes is moderately conserved, and considering the hypothesis that a functional miRNA target site would be conserved across all DENV serotypes, only those target sites common to the four reference sequences were selected. Among the miRNAs that fulfilled these criteria, miR-484 and miR-744 were predicted in our analysis (Fig. 1A) using the RNAhybrid program. The StarMir program predicted the potential binding sites for miR-484 and miR-744 in the DENV 3′ UTR as the target sequences (Fig. 1B). Each of the candidate sites was assigned a logistic probability as a measure of confidence in the predicted site. Interestingly, the location of the target sequence was in a loop of the 3′ UTR known as the 3′-SL that contains the elements known as the 3′ cyclisation sequence (3’CS) and the region upstream of the AUG (3′UAR) (Fig. 1C). Secondary structure was predicted using the Mfold program (http://unafold.rna.albany.edu/?q=mfold). Using the RNAfold webserver we observed that this secondary structure is the natural-mode structure that is most stable for that region in the 3′ UTR of DENV RNA (the free energy of the thermodynamic ensemble was -140.64 kcal/mol) (http://unafold.rna.albany.edu/?q=mfold/rna-folding-form). We then wondered whether the miR-744 and miR-484 binding sites on the 3′ UTR of DENV RNA (Fig. 1C) are accessible in the presence of the SL and hairpin. Using the RNAup webserver (http://rna.tbi.univie.ac.at/cgi-bin/RNAWebSuite/RNAup.cgi) we found that the total free energy of the miR-484-3′ UTR was -8.92 kcal/mol with an opening energy (accessibility) of 5.16 kcal/mol, whereas for the miR-744-3′ UTR the total energy was -7.91 kcal/mol with an opening energy of 8.13 kcal/mol, suggesting that miRNA-3′ UTR binding would proceed spontaneously.

**Fig. 2:**
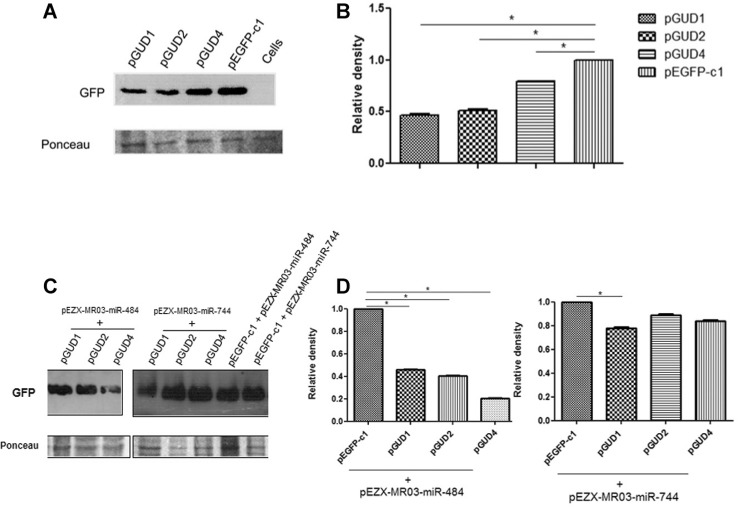
: cellular miRNAs alter the expression of GFP-fused to the 3′ UTR of Dengue virus (DENV) RNA. (A) Vero cells were transfected with pGUD1, pGUD2, pGUD4, or pEGFP-C1 and the GFP expression was determined by western blotting after 24 h, using an anti-GFP antibody; (b) band intensities quantified by densitometry; (C) Vero cells were co-transfected with pGUD1, pGUD2, pGUD4, or pEGFP-C1 and pEZX-mR03-miR-484 or pEZX-mR03-miR-744, and GFP expression was then determined by western blot after 24 h using an anti-GFP antibody; (D) band intensities quantified by densitometry. Data are shown as Median and Range (two way ANOVA). Three independent replicates were performed for each experiment. (*) Statistically significant difference compared to the control (p < 0.05).

*Endogenous miRNAs alter the expression of GFP fused to the 3′ UTR of DENV RNA* - Since our bioinformatics analyses suggested that the DENV 3′ UTR might be targeted by cellular miRNAs, it was important to determine whether the DENV 3′ UTR actually functions as a miRNA target. Based on our findings, the first approach that we used to validate our results was to follow a previously reported strategy (Castillo et al. 2016). The 3′ UTRs of DENV-1, -2, and -4 were individually cloned into the plasmid pEGFP-C1 and the respective constructs, pGUD1, pGUD2, and pGUD4 were obtained. These constructs were subsequently transfected into Vero cells; the empty pEGFP-C1 vector served as the control. Transfection efficiency was determined by fluorescence microscopy and all three plasmids had comparable transfection efficiencies (higher than 50%). We assumed that if endogenous cellular miRNAs that recognise the 3′ UTR of the viral genome exist, a decrease in the GFP expression level compared to pEGFP-C1 should be observed. The GFP expression levels were then assessed by western blotting. Reduced expression of GFP-fused to the 3′ UTR of DENV-1 (pGUD1), DENV-2 (pGUD2), and DENV-4 (pGUD4) versus the control was also observed using the monoclonal anti-GFP antibody (Fig. 2A). Fig. 2B shows the fold change measured by densitometry. A statistically significant inhibitory effect was observed in the expression of GFP fused to the 3′ UTR of DENV-1, DENV-2, and DENV-4 compared with that in the control (pEGFP-C1). To confirm whether among these endogenous miRNAs, miR-484 and miR-744 might have a target site in the DENV 3′ UTR we performed a co-transfection assay using each generated pGUD construct and the plasmids expressing miR-484 and miR-744. Co-transfection of pEGFP-C1 and the miRNA plasmids served as controls. As shown in Fig. 2C-D, significantly reduced GFP expression was observed using miR-484 but unexpectedly with miR-744, GFP reduction was only observed with pGUD1. This might be explained by the difference of the total free energy and opening energy found for miR-744 using the RNAup webserver, which is higher than that for miR-484 and at a lower total energy, and the possibility of an RNA-RNA interaction is greater, as was observed for miR-484. However, the fact that overexpression of miR-744 does not reduce the levels of GFP (Fig. 2D) demands further investigation. In any case, since we observed that Vero cells possess endogenous miRNAs capable of reducing GFP expression (Fig. 2A-B), we wondered whether miR-744 and miR-484 are conserved in humans and monkeys. As a first approach to answer this question, both miR-484 and miR-744 were amplified from HeLa and Vero cells using the same probe. We observed that miR-484 and miR-744 were amplified (data not shown) and although this does not explain the results observed in Fig. 2B, it suggests that the nucleotide sequence of these two miRNAs is conserved in monkeys and humans.

**Fig. 3:**
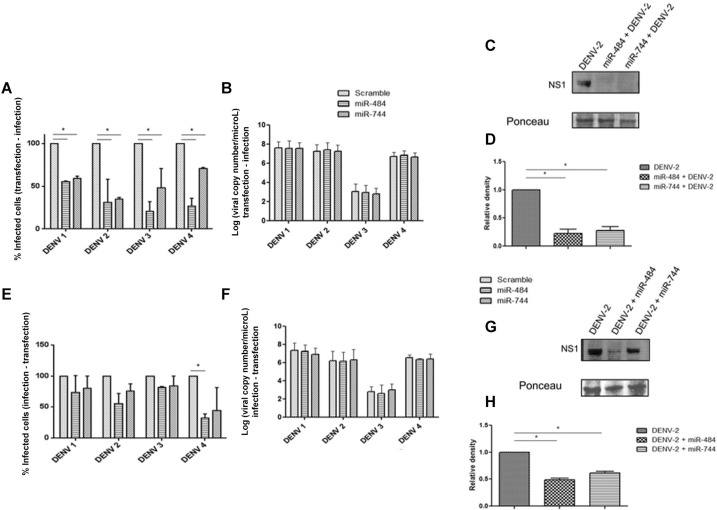
: overexpression of miR-484 and miR-744 modulates Dengue virus (DENV) replication. (A) Vero cells were transfected with pEZX-mR03-miR-484, pEZX-mR03-miR-744, or the empty vector pEZX-mR03 (scramble) and after 24 h, challenged independently with each of the four DENV serotypes at an MOI of 3; (B) quantification of DENV RNA copy number in the supernatants by reverse transcriptase quantitative polymerase chain reaction (RT-qPCR). (C, D) DENV-2 NS1 expression by western blotting for each condition evaluated and the band intensities quantified by densitometry, respectively. Ponceau red was used as the loading control for western blotting, as previously reported (Romero-Calvo et al. 2010, Gilda & Gomes 2013, Rivero-Gutiérrez et al. 2014); (E) Vero cells were first challenged independently with each DENV serotype and then transfected with pEZX-mR03-miR-484, pEZX-mR03-miR-744, or the empty vector pEZX-mR03. The percentage of infected cells was evaluated at 72 hpi by flow cytometry. The data are expressed as the percentage of infected Vero cells compared with those in the scrambled infected Vero cells, defined as 100% infection. Results are shown as Median and Range (two way ANOVA, p < 0.005); (F) Quantification of DENV RNA copy number in the supernatants by RT-qPCR. (G, H) DENV-2 NS1 expression by western blotting for each condition evaluated and band intensities quantified by densitometry, respectively. Ponceau red was used as the loading control for western blotting, as previously reported (Romero-Calvo et al. 2010, Gilda & Gomes 2013, Rivero-Gutiérrez et al. 2014). The data are shown as Median and Range (two way ANOVA). Three independent replicates were performed for each experiment. (*) Statistically significant difference compared to the control (p < 0.05).

*Overexpression of miR-484 or of miR-744 suppresses DENV infection* - Since the DENV 3’UTR contains target sites for miR-484 and miR-744 and since the expression level of GFP fused to the DENV 3’UTR decreased in Vero cells, we hypothesized that both miRNAs might affect DENV infection. Therefore, the effect of miR-484 and miR-744 overexpression on the DENV life cycle was investigated. The lentiviral plasmids pEZX-MR03-miR-484, pEZX-MR03-miR-744, and the pEZX-MR01-control (scrambled) were transfected into Vero cells, followed by challenge with each DENV serotype at an MOI of 3. Alternatively, the cells were first infected with each DENV serotype and then transfected with each plasmid carrying the gene encoding the corresponding miRNA. The percentage of Vero-infected cells, the DENV RNA copy number, and NS1 expression were evaluated 72 hpi under both conditions. As shown in Fig. 3A, when challenge with DENV occurred 24 h after miR-484 or miR-744 overexpression, the percentage of infected cells was significantly reduced for each of the four DENV serotypes compared with that in the scrambled control. Interestingly, the strongest effect was observed with miR-484 overexpression that reduced the infection of DENV-3 and DENV-4 by up to 60%. For DENV-2, a similar effect was observed with either miRNAs. However, when DENV RNA was quantified by RT-qPCR in the Vero culture supernatants, no difference was observed (Fig. 3B). Similar results were observed when the viral RNA within the infected cells was quantified (data not shown). To confirm our finding that miR-484 and miR-744 play a role in the modulation of DENV infection, the expression of DENV-2 NS1 in infected cells was evaluated by western blotting using anti-NS1 antibodies. Overexpression of miR-484 or miR-744 strongly reduced the amount of NS1 in DENV-2-infected cells compared with that in control infected cells (Fig. 3C). Fig. 3D shows the fold change measured by densitometry, indicating a statistically significant decrease in the expression of DENV-2 NS1 upon overexpression of either miRNA.

In contrast, when Vero cells were first challenged with DENV and then transfected independently with pEZX-MR03-miR-484, pEZX-MR03-miR-744, or the pEZX-MR01-control, there was a significant decrease in the percentage of infected cells (less than 50%) only for DENV-4 in the presence of miR-484 (Fig. 3E). Likewise, under these conditions, no change was observed in the number of DENV RNA copies, either in Vero culture supernatants (Fig. 3F) or in the infected cells (data not shown). To verify that both miR-484 and miR-744 indeed affect DENV replication, the level of NS1 expression was determined. DENV-2 NS1 expression was markedly suppressed by miR-484 overexpression, and though the effect of miR-744 overexpression was more modest, NS1 expression was still decreased to about 50% (Fig. 3G). However, as shown in Fig. 3H, when DENV-2 NS1 expression was measured by densitometry, a statistically significant decrease was noted with the overexpression of either miRNA. Taken together, these findings confirm the function of host miR-484 and miR-744 as inhibitors of DENV infection and protein synthesis. Nevertheless, additional studies are required to confirm the antiviral function of these two miRNAs.

**Fig. 4:**
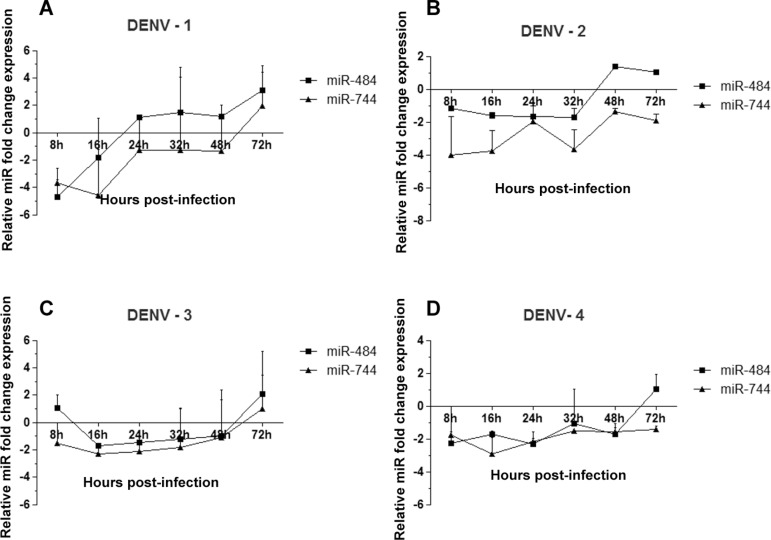
: miR-484 and miR-744 expression levels are downregulated in Dengue virus (DENV)-infected Vero cells. The expression pattern of both miRNAs at different times post-infection in Vero cells infected with DENV-1 to 4 was determined by reverse transcriptase quantitative polymerase chain reaction (RT-qPCR). The results are expressed as the fold change for each miRNA expression level in each sample relative to the mock-infected samples and normalised to 18S rRNA using the 2-ΔΔCt method; (A-D). Data are shown as median and error from three repeated experiments. Three independent replicates were performed for each experiment. (*) Statistically significant difference compared to the control (p < 0.05).

*DENV-1 to -4 infection downregulates the expression of endogenous miR-484 and miR-744 at the early stages of infection* - To better evaluate changes in miR-484 and miR-744 expression, Vero cells were challenged with DENV-1 to -4 at an MOI of 3 or were mock-infected and the expression level of miR-484 and miR-744 was then evaluated at 8, 16, 24, 32, 48, and 72 hpi by RT-qPCR. These stages were considered as early (8-16 h), medium (16-48 h), and late (48-72 h) time-points post-infection. The Ct values were normalised to an uninfected control and to the 18S rRNA (∆∆Ct) to obtain the fold change in expression, according to the manufacturer’s instructions; as previously reported an RQ (relative quantification = 2^-∆∆Ct^) was considered significant at a minimum of two-fold change. The RT-qPCR results show that miR-484 or miR-744 expression was modulated after DENV infection; indeed, as shown in Fig. 4A-D, miR-484 and miR-744 were downregulated at early time points with all DENV serotypes, and depending on the DENV serotype this level was either maintained or increased progressively at the medium time-points, and finally increased progressively at the late time-points post-infection, except for the level of miR-744 in response to DENV-2 and DENV-4 infection. Taken together, these results suggest that all four DENV serotypes can modulate the endogenous expression of miR-484 and miR-744 at different time-points of infection.

**Fig. 5:**
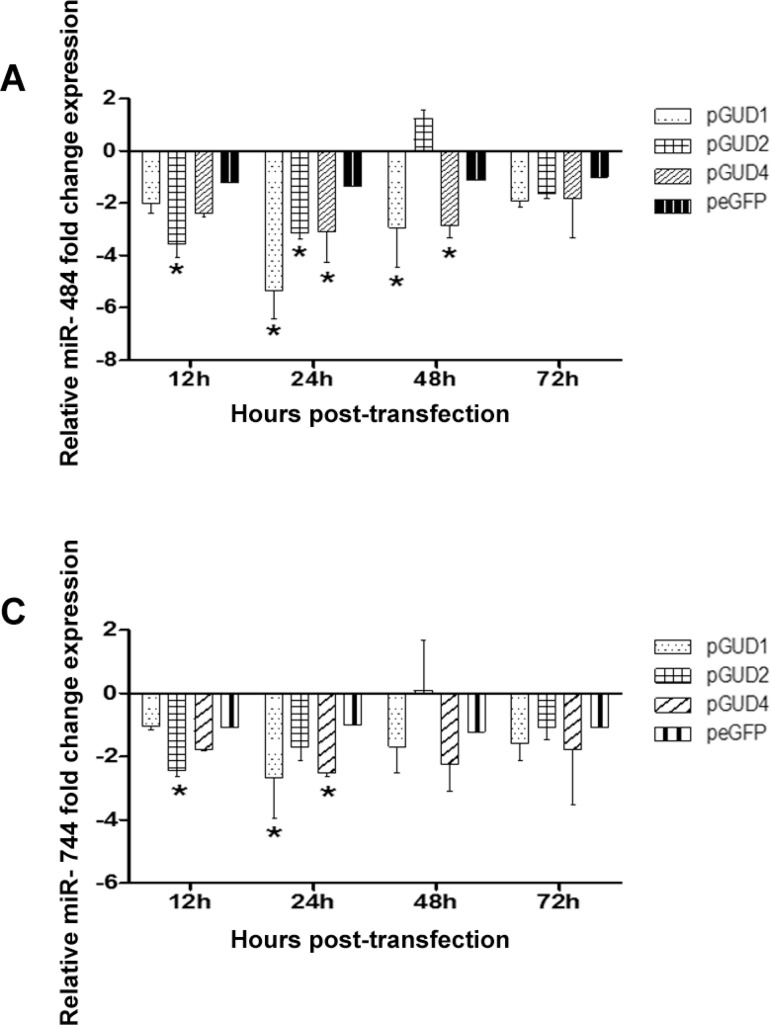
: miR-484 and miR-744 expression is downregulated in Vero cells expressing the Dengue virus (DENV) 3′ UTR. Vero cells were transfected with the pGUD1, pGUD2, or pGUD4 constructs or with the empty vector pEGFP-C1 and their effect on miRNA expression was determined. The expression of miR-484 (A) and miR-744 (B) was evaluated at 12, 24, 48, and 72 hpt by reverse transcriptase quantitative polymerase chain reaction (RT-qPCR) and normalised to the untransfected control and to 18S RNA (2-ΔΔCt). Data from RT-qPCR are shown as median and bars are presented from three independent experiments. (*) Statistically significant difference compared to the control (p < 0.05).

*Endogenous miR-484 and miR-744 expression is downregulated by the DENV 3′ UTR* - It is reported that sfRNA derived from the DENV 3′ UTR is abundantly expressed during infection by all flaviviruses in cultured cells (Lin et al. 2004) and functions as an RNAi suppressor during flavivirus replication; sfRNA has also been shown to interfere in vitro with the processing of the double-stranded RNA (dsRNA) template by Dicer (Schnettler et al. 2012). Because DENV infection downregulates miR-484 and miR-744 expression in Vero cells, we examined whether the DENV 3′ UTR is involved in this process. Thus, Vero cells were transfected with the pGUD1, pGUD2, or pGUD4 constructs, with the pEGFP-C1 empty vector serving as control. The effect of the 3′ UTR on endogenous miR-484 and miR-744 expression was examined at 12, 24, 48, and 72 h post-transfection (hpt) by RT-qPCR, and for the analysis, each time-point was compared with the control (pEGFP-C1) and the levels of significance were determined (*p < 0.05). As shown in Fig. 5A, the 3′ UTR of DENV-1 RNA (pGUD1), DENV-2 RNA (pGUD2), and DENV-4 RNA (pGUD4) induced a greater than 2-fold change, which is statistically significant, in the expression of miR-484, at all-time-points evaluated, except for DENV-2 at 48 hpt (Fig. 5A). Similar results were obtained for miR-744 (Fig. 5B), but since the observed fold change was less pronounced than that with miR-484, possibly because we used the human miR-744 with the model Vero cells of monkey origin. Thus, we wondered if miR-744 and miR-484 are conserved in humans and monkeys. Firstly, to answer this question, we amplified both miR-484 and miR-744 in HeLa cells and Vero cells, by RT-qPCR using the same probe and observed that both miR-484 and miR-744 were amplified (data not shown). Although this finding does not explain the results in Figs 2B and 5B, it allows us to suggest that the nucleotide sequences of these two miRNAs are possibly conserved between monkeys and humans. Interestingly, the 3′ UTR of DENV-1 RNA induced the strongest decrease in miR-484 and miR-744 expression at 24 hpi (Fig. 5A-B).

## DISCUSSION

Several high-throughput studies have provided strong evidence that miRNAs might negatively or positively regulate the viral life cycle or play a critical role in host-virus interactions (Huang et al. 2007). These important features make miRNAs potential therapeutic targets in the treatment of several infectious diseases such as dengue, for which neither therapeutic treatments nor vaccines are currently available. Studies exploring the interaction between DENV and cellular miRNAs will be important for providing insights into the cellular defences against DENV. However, very few reports show the effect of cellular miRNAs on DENV infection or on the changes in miRNA expression during DENV infection. In this study, we focused on the effect of DENV infection on the expression of miR-484 and miR-744 and on the involvement of these two miRNAs on DENV replication when overexpressed. We report that the 3′ UTR of all four DENV serotypes present target sequences for cellular miRNAs, including miR-484 and miR-744. Interestingly, these miRNAs target a sequence localised in the 3′-SL of the 3′ UTR of DENV RNA. Since this 3′-SL contains the 3′CS and 3′UAR elements that are critical for efficient host and viral protein recruitment involved in viral replication, we suggest that the interaction of the 3′ UTR with miR-484 or miR-744 might alter the viral life cycle. This notion is strengthened by the results obtained in Vero cells expressing GFP-fused to the 3′ UTR of DENV plasmids that showed decreased GFP expression (Fig. 2A-B). However, further studies are needed to confirm this hypothesis, since overexpression of GFP-fused to the 3′ UTR of the DENV constructs in the presence of a plasmid containing the miR-744 gene did not yield the same results, except with pGUD1, i.e. we did not observe decreased GFP expression (Fig. 2C-D). However, it should be considered that although our *in silico* prediction of miR-484 and miR-744 interaction with DENV RNA relies on sequence complementarity and site homology, the seed sequence complementarity (shaded nucleotides) is lower for miR-744 than that for miR-484 (black circles) as shown in Fig. 1B, indicating that these two miRNAs have different sequences, including the seed sequence present in the first nucleotides from the 5′ end.

It has been shown that miR-484 and miR-744 may be expressed in different human cells including macrophages and dendritic cells (our unpublished observations), which are both DENV target cells, and that their expression can be regulated by external factors that alter cell homeostasis, such as diseases (Tan et al. 2015). However, despite the different studies on the expression of these miRNAs, thus far, no specific function has been attributed to either miRNA, but miR-744 was shown to enhance the IFN-I signalling pathway by targeting protein tyrosine phosphatase 1B, a ubiquitously expressed phosphatase, in primary human renal meningeal cells (Zhang et al. 2015).

Since miR-484 and miR-744 overexpression suppresses DENV-2 protein production such as NS1, we suggest that both miRNAs might affect DENV replication, but simultaneously, DENV infection decreases the expression of miR-744 and miR-484. Although the mechanisms through which miR-484 and miR-744 inhibit DENV replication remain uncertain, the DENV 3′ UTR is known to contain conserved sequences involved in viral RNA circularisation and host-protein interaction. Consequently, such an effect might be associated with interference in these events. For example, PTB is reported to interact with the 3′ UTR of DENV RNA and is required for efficient DENV propagation in Vero cells (de Nova-Ocampo et al. 2002). Furthermore, we recently reported that miR-133a inhibits DENV replication by decreasing PTB expression (Castillo et al. 2016), a direct and functional target of miR-133a. Interestingly, the target sites of miR-133a, miR-484, and miR-744 are located in a single region, highlighting a ‘hotspot’ of potential MRE. The interaction of these miRNAs with the 3′ UTR of DENV RNA might lead to failure in the recruitment of PTB, thus affecting the circularisation of the viral genome, with consequences on virus replication or RNA translation. Our data are consistent with those of a previous report showing that cellular miRNAs suppress/inhibit DENV replication (Wen et al. 2015). Furthermore, antisense oligomers or small interfering RNAs directed against target sequences in the 3′ UTR of DENV RNA are also known to inhibit virus replication and translation, presumably by blocking either the RNA-RNA interactions by steric interference, or the RNA-protein interaction complexes involved in the synthesis or translation of viral RNA (Holden et al. 2006). Similar results were observed using a related strategy for the avian leucosis virus subgroup J (ALV-J) (Wang et al. 2013), where the authors showed that the activity of the luciferase-reporter gene carrying the 5′ and 3′ UTR of ALV-J decreased with the host-encoded gga-miR-1650 that interacts with the 5′ UTR. The demonstration that host miR-484 and miR-744 share a target site in the 3′ UTR of the RNA in all four DENV serotypes suggests that these miRNAs might be part of the host antiviral response against DENV. However, it is necessary to emphasize that the anti-DENV activity of miR-744 was much stronger compared to the activity observed with miR-744. Notably, this is an unexpected result since both miRNAs share some degree of sequence homology, although the miR-484 seed region shares complete homology with the 3′ UTR of the DENV recognition site. In line with our results, Wu et al. (2014) also recently reported that DENV-2 infection decreases miR-223 expression in Vero cells, but that overexpression of miR-223 suppresses DENV-2 replication, suggesting that miR-223 may be present as an antiviral factor against DENV-2. Furthermore, our results gain importance because miR-744 was reported to direct the post-transcriptional regulation of TGF-β1, which is crucial in inflammation (Martin et al. 2011), and this cytokine is reported to be involved in severe dengue disease (Chen et al. 2009). In addition, a large body of evidence has indicated that TGF-β1 gene polymorphisms are associated with increased susceptibility to dengue haemorrhagic fever and higher virus load, and high TGF-β1 production has been linked to protection or a mild clinical outcome of dengue infection (Chen et al. 2009).

Although our results suggest that miR-484 and miR-744 possess antiviral activity, we also observed that both DENV infection and the expression of the 3′ UTR of DENV RNA can downregulate miR-484 and miR-744. Although it is still not clear how DENV promotes miR-484 and miR-744 downregulation, previous findings have identified the pathway by which DENV downregulates the expression of cellular miRNAs. Indeed, NS4B from all 4 DENV serotypes appears to inhibit the processing of Dicer, a protein involved in miRNA biogenesis of dsRNA (Kakumani et al. 2013). Similar behaviour has been reported for other viral proteins such as HIV-1 Nef, a regulatory and accessory protein that interacts with the RNAi pathway protein, Ago2, thereby suppressing miRNA silencing concentrated in the multivesicular bodies where HIV-1 actively replicates (Aqil et al. 2013). Interestingly, several recent reports have provided evidence that certain non-coding RNAs may function as competing endogenous RNAs (ceRNAs) in modulating the concentration and biological functions of miRNAs. These ceRNAs generally share miR-response elements with the transcripts of several important genes and prevent these mRNAs from being degraded. In accordance with these results, it was recently proposed that HBV mRNA acts as a ceRNA for miRNA-15a to regulate TGF-signalling, which contributes to the development of HBV-related hepatocellular carcinomas (HCCs) (Liu et al. 2015). Based on these results and because it was demonstrated that the West Nile virus (WNV) sfRNA suppresses the siRNA- and miR-induced RNAi pathway (Schnettler et al. 2012), we suggest a role for DENV RNA, through its 3′ UTR or sfRNA, that would act as a sponge for the hybridisation of endogenous miRNAs, and as a potential mechanism to reduce their expression; such a mechanism was demonstrated for HBV mRNAs possessing a miR-15a/16-complementary site that acts as a sponge to bind and sequester endogenous miR-15a/16 (Liu et al. 2013).

We propose that the interaction of viral RNA with the deregulated RNA-induced silencing complex (RISC) containing the mature miRNAs could prevent genome circularisation, thus affecting viral RNA translation. This could explain the reduction in the percentage of infected cells observed at 72 hpi, compared to that in the control (scrambled) cells, but only when either miR-744 or miR-484 were first overexpressed, whereas no significant effect was observed when the cells were first infected, and at 24 hpi either miRNA was overexpressed with no reduction in DENV RNA in either strategy. This is interesting since the expression of miR-744 and miR-484 was decreased during the first 48 hpi but later, their expression was increased mainly at 72 hpi in Vero cells infected with DENV-1 or DENV-3. Why the expression of these two miRNAs increases with a delay only in response to DENV infection but not when the 3′ UTR fused downstream of the GFP ORF was overexpressed, is unknown. We suggest that this effect is due to an immune interaction caused by DENV infection; e.g., in natural killer cells, TLR7 stimulation was reported to induce the expression of miR-744 (Voynova et al. 2015). Further related studies are being performed in our laboratory to examine and confirm these possible mechanisms of miR-484 and miR-744 action in DENV infection, using primary human cells, such as macrophages/dendritic cells, or in patients with dengue. However, further studies will be required to lend weight to this possibility and to determine whether miR-484 and miR-744 are possible therapeutic targets for all DENV serotypes, even more so if we consider that the present study was carried out in monkey cells. In summary, we propose that DENV can escape the antiviral activity of miR-484 and miR-744, downregulating their expression, and simultaneously, that miRNAs target an unknown cellular factor that promotes DENV replication. Therefore, our results imply that DENV infection modulates host miR expression for their own benefit.

miR-484 and miR-744 can be considered as two possible restriction host factors against DENV infection, but the virus might have evolved to resist inhibition by endogenous human miRNAs during productive replication. This finding suggests that DENV RNA is a target of cellular miRNAs. Furthermore, this study contributes to a better understanding of the relationship between host miRNAs and DENV, and recommends further studies to decipher the biological functions of this interaction to develop a therapy based on these two miRNAs for the control of infection and treatment of dengue illnesses.
